# “Adenomatous Polyps Of The Gallbladder” Adenomas oF the Gallbladder

**DOI:** 10.1155/1991/59324

**Published:** 1991

**Authors:** Attilio Maria Farinon, Antonio Pacella, Francesco Cetta, Mario Sianesi

**Affiliations:** 1 Chair of Surgical Pathology II University of Rome Italy; 2 Chair of Surgical Pathology University of Siena Italy; 3 Chair of Surgical Pathology University of Parma Italy; 4 Dipartimento di Chirurgia II Università di Roma Via O. Raimondo Roma 1-00173 Italy

## Abstract

The finding of adenomatous polyps of the gallbladder is a rare occurrence and an unusual clinical problem.

Among 2,145 patients who underwent cholecystectomy for gallbladder disease only 9 (0.4 per cent) presented with adenomatous polyps. There were 6 women and 3 men, aged 17 to 70 years. Preoperative ultrasonographic diagnosis was made in only 1 of 7 patients with gallstones, in contrast polypoid lesions within a gallbladder without stones were easily confirmed by both ultrasonography and oral cholecystography in the remaining 2 patients. All polyps were 1.0 cm or less in size and without histologic evidence of malignant change. The clinical significance of this rare condition is discussed, with particular reference to a possible role in development of gallbladder carcinoma. Surgical treatment should be advocated regardless of clinical manifestation when the polyp exceeds 1.0 cm in diameter or rapid growth of the lesion is seen on ultrasonographic follow-up examinations.


*HPB Surgery*, 1991, Vol. 3, pp. 251–258
Reprints available directly from the publisher
Photocopying permitted by license only

© 1991 Harwood Academic Publishers GmbH
Printed in the United Kingdom

### "ADENOMATOUS POLYPS OF THE GALLBLADDER" ADENOMAS OF THE GALLBLADDER

ATTILIO MARIA FARINON and ANTONIO PACELLA
*Chair of Surgical Pathology, II University of Rome*

FRANCESCO CETTA
*Chair of Surgical Pathology, University of Siena*

MARIO SIANESI
*Chair of Surgical Pathology, University of Parma, Italy*

*(Received 8 August 1990)*

The finding of adenomatous polyps of the gallbladder is a rare occurrence and an unusual clinical problem.

Among 2,145 patients who underwent cholecystectomy for gallbladder disease only 9 (0.4 per cent) presented with adenomatous polyps. There were 6 women and 3 men, aged 17 to 70 years. Preoperative ultrasonographic diagnosis was made in only 1 of 7 patients with gallstones, in contrast polypoid lesions within a gallbladder without stones were easily confirmed by both ultrasonography and oral cholecystography in the remaining 2 patients. All polyps were 1.0 cm or less in size and without histologic evidence of malignant change. The clinical significance of this rare condition is discussed, with particular reference to a possible role in development of gallbladder carcinoma. Surgical treatment should be advocated regardless of clinical manifestation when the polyp exceeds 1.0 cm in diameter or rapid growth of the lesion is seen on ultrasonographic follow-up examinations.

KEY WORDS: gallbladder, polyp, adenoma

### INTRODUCTION

Recent interest in polypoid lesions of the gallbladder has arisen from the wide application of ultrasonography, which can provide a precise demonstration of them and readily detect any changes[1,2,3]. Nevertheless, their nature is difficult to define because of the wide array of conditions that these polyps may represent, including adenomatous polyps, hyperplastic polyps, cholesterol polyps, inflammatory epithelial proliferations, adenomyomas, and carcinomas.

Until recently, confusion often followed the discovery of polyps within the gallbladder, contributed to by the internist, who generally ignored them, the radiologist, who tended to minimize their importance, and the pathologist, whose reports could confuse everyone[4].

Address for correspondence: Prof. A.M. Farinon, Dipartimento di Chirurgia, II Università di Roma, Via O. Raimondo 1–00173 Roma, Italia

However, the finding of adenomatous polyps of the gallbladder remains a rare occurrence and an unusual clinical problem.

### MATERIALS AND METHODS

Between 1986 and 1989, 2,145 patients underwent cholecystectomy for gallbladder disease in three different university surgical institutions (Surgical Pathology, II University of Rome; Surgical Pathology, University of Siena; 2nd Surgical Pathology, University of Parma). All the gallbladders were opened following excision and macroscopic characteristics of the gallbladder wall were recorded. In order to avoid autolytic changes in the mucosa, the surgical specimens with parietal alterations were immediately fixed in 10 per cent formaldehyde; 4-$\mu$m sections of paraffin-embedded specimens were stained with hematoxylin and eosin, and in some of the cases sections were prepared with special stains, including alcian blue and oil red O.

Adenomatous polyps of the gallbladder were present in 9 patients (0.4 per cent); cholesterol polyps, inflammatory polyps, and adenomyomas were not considered in this study.

There were 6 women and 3 men (F/M ratio, 2:1) aged 17 to 70 years (mean age: 48.2 years).

Clinical data and preoperative diagnosis were correlated with the pathologic characteristics of the polyps.

### RESULTS

Cholecystectomy was performed because of an ultrasonographic diagnosis of gallstones in 7 patients. Two patients underwent operation for a gallbladder containing a single polyp, but no stones, detected by both ultrasonography and oral cholecystography (Figure 1). Among the 7 patients with lithiasis, gallstones masked the presence of polypoid lesions in 6 and the polyp was correctly diagnosed before surgery only in one patient with microlithiasis of the gallbladder.

The two patients with a preoperative diagnosis of a gallbladder polyp were both young: a 17 year old male and a 29 year old female. In the former, surgery was indicated because of recurrent right upper quadrant and epigastric pain, in the latter who was asymptomatic, the ultrasonographic follow-up showed rapid growth of the lesion, from 0.6 to 1.0 cm within six months.

All symptomatic patients were relieved of symptoms following cholecystectomy. The polyps were single in all but one patient, whose gallbladder contained three adenomas with numerous gallstones. The sizes of the eleven lesions recovered were 1.0 cm or less in diameter, and 7 were 0.5 cm or less. Macroscopically, all were pedunculated. Based on histopathologic characteristics, the adenomas, as in the intestine, were classified into three types, according to Albores-Saavedra and Henson[5]: tubular, papillary, or mixed. The tubular type was the most common (6 of 11), whereas the papillary type was recognized in 3 lesions, and a mixed tubular and papillary pattern characterized the remnant 2 polyps. No malignant changes were found within the polypoid lesions (Figure 2) and only low-grade (i.e. mild and moderate) dysplastic appearances were observed.

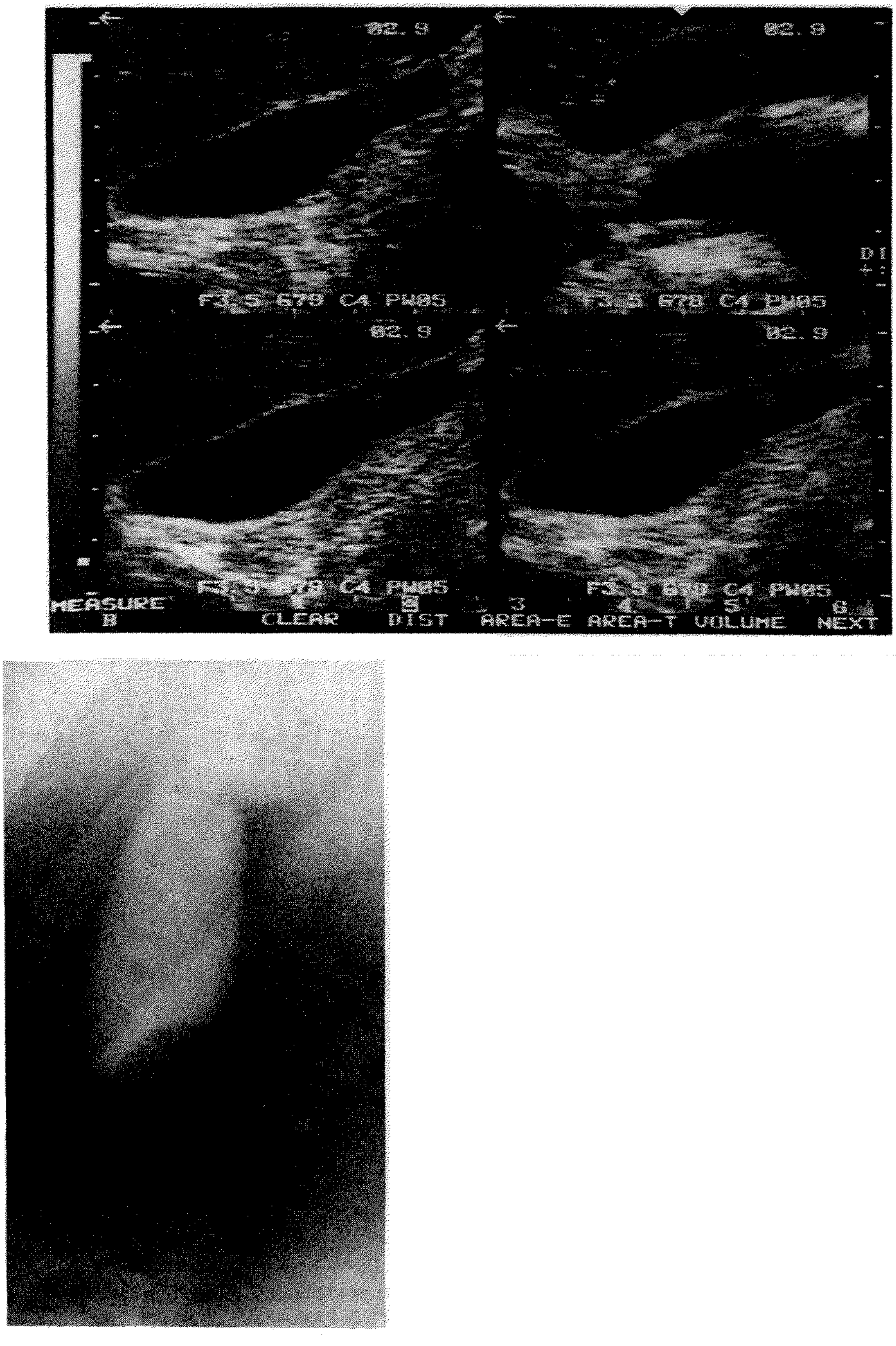

**Figure 1** M.R., 17 years, male — Ultrasonographic findings (above), and cholecystographic and gross pathologic appearances (below) of a 0.4 cm adenomatous polyp of the gallbladder are presented.

(See Colour Plate at back of issue)

**Figure 2** S.F., 70 years, male — A) the removed gallbladder contains a 0.5 cm polypoid lesion; B) histological appearances of the polypoid lesion, with a relatively broad stalk, show its adenomatous nature with a tubular structure (H & E. × 55); C) glands of variable width, at time characterized by enlarged lumen, are evident in a close view of the previous picture (H & E. × 125); D) at higher magnification, a slight depletion of mucus and scattered features of mild dysplasia are observable (H & E. × 310).

(See Colour Plate at back of issue)

### DISCUSSION

Adenomatous polyps of the gallbladder, often reported in the literature as papillomas, polyps, and adenomas, are benign epithelial tumors. Their occurrence is routinely described as rare and their true incidence is unknown, although in published reviews their operative incidence is less than 1 per cent of cholecystectomies[5]. Majeski[6] in a complete review of the surgical pathology and autopsy data from the Medical University of South Carolina, found only three cases of papillary adenomatous polyps in fifteen years. Albores-Saavedra and Henson[5] suggested an incidence of 0.5 per cent in their material of 4,000 cholecystectomy specimens. Muto *et al.*[3] reported 3 adenomas (1.4 per cent) in a series of 207 polypoid lesions of the gallbladder. Koga *et al.*[2] found only 1 adenoma among 40 (2.5 per cent) polypoid lesions of the gallbladder recovered from 411(0.2 per cent) patients who underwent cholecystectomy. This apparent rarity could be related to the latent nature of these lesions incidentally found in the surgically removed gallbladders[3,7].

Adenomas of the gallbladder, defined by the WHO classification[8] as benign glandular tumors composed of cells resembling biliary tract epithelium, may be histologically classified into papillary and non-papillary types, according to Christensen and Ishak[9] and Weedon[10]. More recently, Yamamoto *et al.*[14], in an effort to describe the characteristics of the epithelium composing the polypoid lesions, distinguished two types of adenoma: the ordinary-type adenoma, as that defined by the WHO classification, and the metaplastic-type adenoma, characterized by metaplastic epithelial proliferation, which could play an important role in the development of gallbladder carcinoma. However, a more simple classification for clinical purposes is that proposed by Albores-Saavedra and Henson[5], who divided adenomas in a similar way to those arising from intestine, i.e., tubular, papillary, and mixed type.

Clinically, adenomatous polyps and other polypoid lesions of the gallbladder are usually detected in the course of routine surveys during investigation for other gastrointestinal disease. It is generally believed that a polyp itself is asymptomatic and the clinical manifestation is due to the accompanying cholecystitis, cholelithiasis or both[2,3]. Moreover, in the symptomatic patients without gallstones it is uncertain how and to what extent adenomatous polyps contribute to symptoms. Two possible mechanisms may explain dyspeptic symptoms or attacks of biliary colic. Firstly, the symptoms may be caused by a prolapse of the adenoma into Hartmann's pouch and then spontaneously reducing itself[12], secondly, the clinical presentation may be related to the breaking off of a portion of adenoma, which lying free into the lumen of the gallbladder could obstruct the cystic duct[12,13]. This latter explanation, the more acceptable, could account for the symptoms of the youngest patient in our series.

The most useful methods for the detection of these lesions are undoubtedly ultrasonography and oral cholecystography. Unfortunately, these diagnostic methods, which demonstrate the presence of polypoid lesions when not masked by gallstones, do not allow the preoperative determination of their true nature, which can be defined only by histopathologic examination[3]. However, on the basis of ultrasonographic findings a differentiation between neoplastic and cholesterol polyp may be possible: the former being characterized by a loose, and the latter a compact, echo-pattern. It is impossible to differentiate neoplastic polyps from adenomyomas, by their echo-patterns because of their similar vascularization[14].

Irrespective of these considerations, the ultrasonographic determination of the polyp size is needed to interpret the clinical significance of these polypoid lesions.

In spite of limited knowledge about pathologic features and potentialities of adenomatous polyps of the gallbladder, it has been suggested that the likelihood of malignancy increases with their size and that a malignant condition should be considered whenever they exceed 1.0 cm in diameter[2,3,7]. Tsuchiya and Uchimura[15], in a collective review of small polypoid lesions of the gallbladder from 15 japanese institutions, reported a 6 per cent incidence of carcinoma in lesions less than 1.0 cm, while the incidence of carcinoma increased to 37.5 per cent in those 1.0–2.0 cm. Moreover, Kozuka *et al.*[11], as a result of the histopathologic examination of 1,605 cholecystectomy specimens found, among 18 adenomas, 11 benign lesions 1.2 cm or less in diameter and 7 with malignant changes 1.2 cm or more in diameter. Thus, as suggested by Muto *et al.*[3] the size of the polyp within the gallbladder may represent a crucial feature in distinguishing benign from malignant lesions. In other words, gradual increase in size of the lesions is matched by advance of their malignant changes (Table 1). Even if Ruiz *et al.*[16] failed to demonstrate correlation between size of the neoplastic lesion and extension, the study of Koga *et al.*[2] clearly indicated a close correlation of depth of invasion and extent of spread to the enlargement of the lesion.

**Table 1** Relationship of size to carcinoma risk in gallbladder adenomas

| *Author* | *Benign adenoma N.* | *Diameter(mm)* | | *Adenoma with malignant change Diameter (mm)* | *N.* |
|---|---|---|---|---|---|
| Kozuka *et al.*[7] | 11 | 5.5±3.1 | | 17.6±4.4 | 7 |
| Muto *et al.*[3] | 3 | <10 | | >10 | 30 |
| Koga *et al.*[2] | 1 | <10 | (6–10)* | >10 | 8 |
| Present series | 11 | 5.8±2.2 | | – | – |

* = 6 to 10 mm adenoma may be a cancer

The past controversies over the malignant potential of these lesions are now overcome and malignant changes of benign epithelial lesions are thought to be not infrequent[11]. Tabah e Mc Neer[17] reported 3 carcinomas among 4 papillomas, and Azaki e Tahara[18], from an extensive review of 99 benign epithelial polypoid lesions, found 15 lesions (15.2 per cent) with malignant changes. The study of Kozuka *et al.*[7] most conclusively supports the adenoma-carcinoma sequence in the gallbladder, as for most carcinomas in the digestive tract. Their study showed a 19 per cent (15 out of 79) incidence of the adenomatous residue in invasive carcinomas, in addition to the presence of carcinomatous foci in 7 (38.8 per cent) out of 18 adenomas. Recently, Yamamoto *et al.*[11] suggested the possibility that metaplastic changes could be responsible for the precancerous characteristics of adenomas; 7 out of 14 metaplastic-type adenomas presented foci of atypical glandular proliferation with structural and cellular atypia.

From a practical point of view, the treatment of polyps of the gallbladder has not been standardized, probably because of the relative rarity of adenomas and limited knowledge of their clinical significance. Our data, as those by others[2,3,15], support the following policy in the treatment of patients with polypoid lesion of the gallbladder.

Early cholecystectomy should be recommended whenever these lesions are associated with gallstones, albeit surgical decision making in these cases is more related to the presence of cholelithiasis, even asymptomatic, than the finding of polyps which are often not detected preoperatively.

The major problem is whether cholecystectomy should be advocated in the management of either symptomatic or asymptomatic of the patients with an ultrasonographically detected polyp in the absence of gallstones. Our opinion is that symptomatic lesions should be removed regardless of size, because generally the patients are relieved of symptoms after cholecystectomy. In contrast, in asymptomatic patients with a lesion less than 0.5 cm in diameter surgery may be deferred and ultrasonographic follow-up at interval of three months should be established. If no growth is observed it may be presumed that the lesion is likely to be benign. Growth of polyps on planned ultrasonographic examinations suggests the possibility of malignancy and justifies cholecystectomy. Polyps exceeding 1.0 cm in diameter should be surgically removed regardless of clinical presentation[3] and the patients with these lesions should be considered candidates for elective surgery for gallbladder cancer[2].

### *References*

1. Cooperberg, P. and Golding, R.H. (1982) Advance in ultrasonography of the gallbladder and biliary tract. *Radiological Clinics of North America*, **20**, 611–633
2. Koga, A., Watanabe, K., Fukuyama, T., Takiguchi, S. and Nakayama, F. (1988) Diagnosis and operative indications for polypoid lesions of the gallbladder. *Archives of Surgery*, **123**, 26–29
3. Muto, Y., Yamada, M., Uchimura, M. and Okamoto, K. (1987) Polypoid lesions of the gallbladder. *Italian Journal of Surgical Sciences*, **17**, 171–178
4. Sawyer, K.C. (1970) The unrecognized significance of papillomas, polyps, and adenomas of the gallbladder. *American Journal of Surgery*, **120**, 570–578
5. Albores-Saavedra, J. and Henson, D.E. (1986) *Tumors of the gallbladder and extrahepatic bile duct*, pp 17–25. Washington: US Armed Forces Institute of Pathology
6. Majeski, J.A. (1986) Polyps of the gallbladder. *Journal of Surgical Oncology*, **32**, 16–18
7. Kozuka, S., Tsubone, M., Yasui, A. and Hachisuka, K. (1982) Relation of adenoma to carcinoma in the gallbladder. *Cancer*, **50**, 2226–2234
8. Gibson, J.B. (1978) *Histological typing of tumours of the liver, biliary tract and pancreas*, pp 31–34. Geneve: World Health Organization
9. Christensen, A.H. and Ishak, K.G. (1970) Benign tumors and pseudotumors of the gallbladder review of 180 cases. *Archives of Pathology and Laboratory Medicine*, **90**, 423–432
10. Weedon, D. (1984) *Pathology of gallbladder*, pp 195–222. New York: Masson Publishing USA
11. Yamamoto, M., Nakajo, S. and Tahara, E. (1988) Histological classification of epithelial polypoid lesions of the gallbladder. *Acta Pathologica Japanica*, **38**, 181–192
12. McGregor, J.C. and Cordiner, J.W. (1974) Papilloma of the gallbladder. *British Journal of Surgery*, **61**, 356–358
13. Kane, C.F., Brown, C.H. and Hoerr, S.O. (1952) Papilloma of the gallbladder. *American Journal of Surgery*, **83**, 161–165
14. Sianesi, M., Rossi, A., Baratta, V. and Farinon, A.M. (1987) Microformazioni ecogene parietali della colecisti. Caratterizzazione ecotomografica. *Acta Chirurgica Italica*, **43**, 1148–1153
15. Tsuchiya, K. and Uchimura, M. (1986) Collective review of 503 cases of small polypoid lesions (less than 20mm in maximum diameter) of the gallbladder. Size distribution in various diseases and that of depth of carcinomatous invasion. *Japanese Journal of Gastroenterology*, **83**, 2086–2087
16. Ruiz, R., Teyssou, H., Fernandez, N., Carrez, J.P., Gortchakoff, M., Manteau, G., Ter-Davtian, P.M. and Tessier, J.P. (1980) Ultrasonic diagnosis of primary carcinoma of the gallbladder: a review of 16 cases. *Journal of Clinical Ultrasound*, **8**, 489–495

17. Tabah, E.J. and McNeer, G. (1953) Papilloma of the gallbladder with in situ carcinoma. *Surgery*, **43**, 57–71
18. Azaki, O. and Tahara, E. (1975) A case report of early cancer arised from papillary adenoma of gallbladder: statistical observation of benign tumors of gallbladder in Japan. *Gan No Rinsho,* **21**, 220–229

(*Accepted by S. Bengmark 8 August 1990)*

### INVITED COMMENTARY

The authors provide a careful review of a rare lesion. Gallbladder polyps are almost always cholesterol polyps and any type of gallbladder polyp may cause biliary colic, presumably because of sloughing of part of the polyp, as the authors state. Few would disagree that rapidly growing polyps or polyps over 1cm should be removed even in asymptomatic patients, although because of the rare nature of the lesion conclusive evidence that this is necessary will be difficult to obtain. Also because of the rarity of the lesion one could not recommend screening of populations to detect such lesions.

Steven M. Strasberg
Toronto, Canada

